# IPSS-M outperforms IPSS-R in prognostic stratification and guides effective interventions for very High-Risk myelodysplastic syndrome patients undergoing allogeneic hematopoietic stem cell transplantation

**DOI:** 10.1007/s12672-025-03155-1

**Published:** 2025-07-11

**Authors:** Huixian Wu, Shuang Li, Jun Yang, Yu Cai, Huiying Qiu, Chongmei Huang, Yin Tong, Kun Zhou, Jiahua Niu, Xinxin Xia, Ying Zhang, Xiaowei Xu, Chang Shen, Baoxia Dong, Liping Wan, Xianmin Song

**Affiliations:** Department of Hematology, Shanghai General Hospital, Shanghai Jiaotong University School of Medicine, No. 100 Haining Road, Shanghai, 200080 China

**Keywords:** Myelodysplastic syndrome (MDS), Allogeneic peripheral blood stem cell transplantation, Revised International Prognostic Scoring System (IPSS-R), Molecular International Prognostic Scoring System (IPSS-M), Prophylactic Intervention

## Abstract

Allogeneic hematopoietic stem cell transplantation represents a curative modality for patients with myelodysplastic syndrome (MDS), yet relapse risk persists. The prognostic performances of the Revised International Prognostic Scoring System (IPSS-R) and the Molecular International Prognostic Scoring System (IPSS-M) for 129 MDS patients undergoing allogeneic peripheral blood stem cell transplantation (allo-PBSCT) were evaluated. Using IPSS-M, 29.5% of patients were reclassified to a higher risk and 14.0% to a lower risk compared to IPSS-R. The two-year recurrence-free survival (RFS) and overall survival (OS) post-transplant were similar across IPSS-R groups, but significantly lower in the very-high risk category with IPSS-M (*P* = 0.005 for RFS; *P* = 0.014 for OS). Multivariate analysis revealed that patient age (*P* = 0.009, *P* = 0.017), very-high risk category in IPSS-M (*P* = 0.003, *P* = 0.001), and KPS score (*P* = 0.034, *P* = 0.055) were independent factors for OS and RFS. Only very-high risk category in IPSS-M was the independent factor affecting cumulative incidence of relapse (*P* = 0.003). Prophylactic interventions for relapse significantly decreased the risk of relapse (*P* = 0.003) and increased the survival of patients in the very-high risk category (*P* = 0.002 for OS, *P* = 0.006 for RFS). The study suggested that the IPSS-M system could identify the high relapse risk patients post-transplant who may benefit from early prophylactic interventions.

**Supplementary Information:**

The online version contains supplementary material available at 10.1007/s12672-025-03155-1.

## Introduction

Myelodysplastic syndrome (MDS) comprise a diverse array of clonal hematopoietic stem cell disorders characterized by morphological dysplasia, ineffective hematopoiesis, peripheral blood cytopenia, and a predisposition to progress to acute myeloid leukemia (AML) [1]. Allogeneic hematopoietic stem cell transplantation (allo-HSCT) remains a critical therapeutic strategy for MDS patients, offering a potential cure, particularly in younger individuals [2]. However, the high incidences of transplant-related complications and mortality cannot be overlooked. In the clinical management of MDS, risk stratification is pivotal in guiding therapeutic decisions. The Revised International Prognostic Scoring System (IPSS-R) has been a robust tool for stratifying MDS, incorporating bone marrow blast percentage, cytopenia, and cytogenetic abnormality, while it does not account for molecular insights [3, 4]. With the advent of next-generation sequencing (NGS), a wealth of molecular data has emerged, leading to the development of the Molecular International Prognostic Scoring System (IPSS-M) by the International Working Group for Prognosis in MDS (IWGPM) in 2022 [5, 6]. This model integrates hematological and cytogenetic data with mutations across 31 genes implicated in MDS, offering enhanced prognostic precision. Studies have consistently shown that IPSS-M surpasses IPSS-R in prognostic accuracy for various clinical outcomes [7–9]. In this study, we evaluated the prognostic performances of IPSS-R and IPSS-M for patients with MDS undergoing allogeneic peripheral blood stem cell transplantation (allo-PBSCT) and investigated whether prophylactic intervention could improve the post-transplant survival based on IPSS-M risk category.

## Materials and methods

### Patients

A consecutive cohort of 134 patients was diagnosed with MDS according to the 2016 World Health Organization (WHO) criteria [10] and underwent allo-PBSCT at our center between June 2016 to February 2023. Risk stratification was performed at the time of MDS diagnosis using both the IPSS-R and the IPSS-M [3, 6]. Cytogenetic analyses at diagnosis were performed using standard techniques of banding, in situ hybridization, and NGS.The study protocol adhered to the Declaration of Helsinki principles and received approval from the institutional review board of Shanghai General Hospital. Written informed consent was obtained from all participants or their legal guardians. Follow-up data were current as of June 1, 2024.

### Transplant procedures

**Fig. 1:**
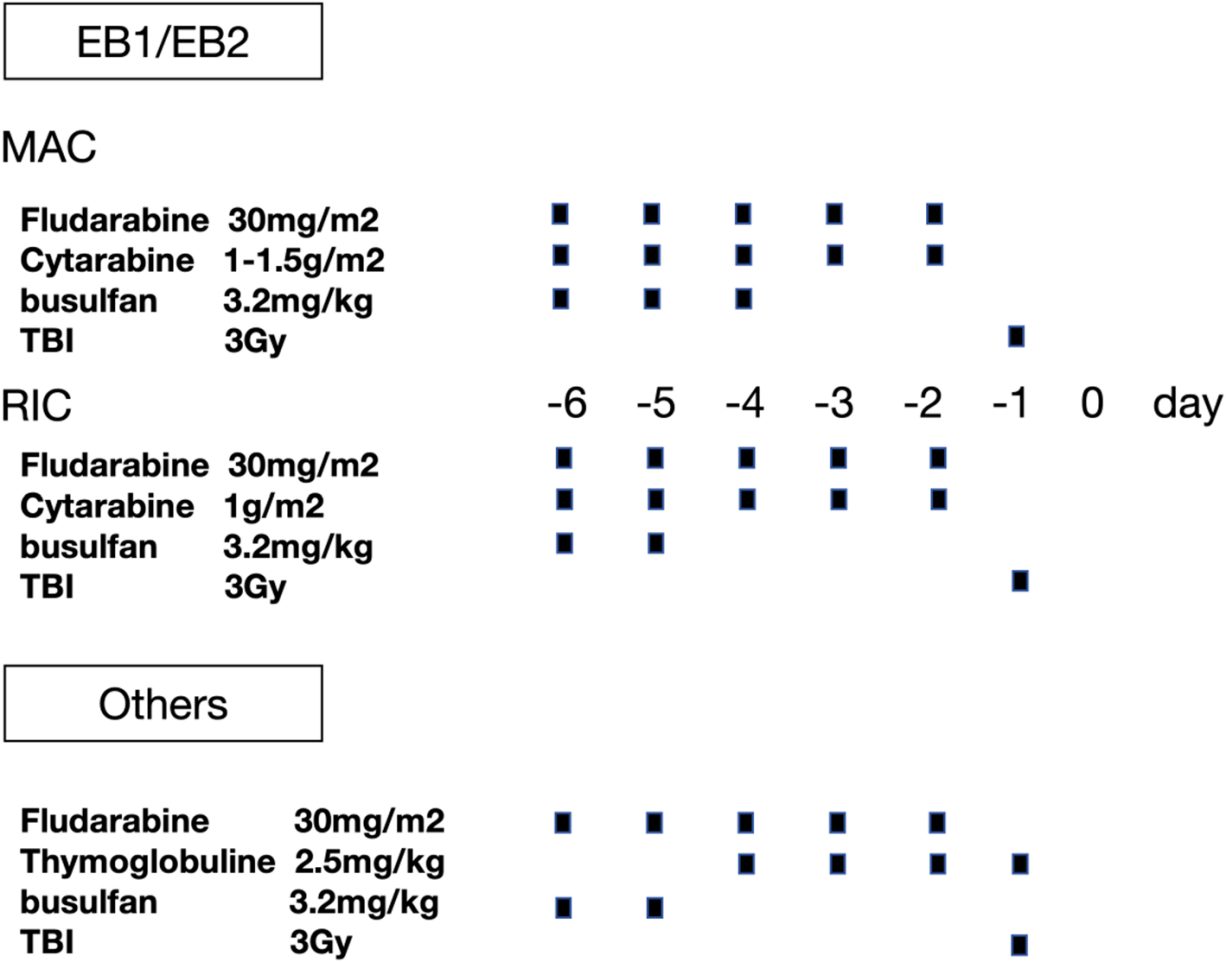
The conditioning regimens. MAC myeloablative conditioning; RIC, reduced-intensity conditioning; TBI, total body irradiation

Donors were selected based on high-resolution human leukocyte antigen (HLA) typing at the HLA-A, HLA-B, HLA-C, HLA-DRB1, and HLA-DQB1 loci. Family member donors included HLA-matched sibling (MSD) and haploidentical donors. Unrelated donors (URDs) were from the China Marrow Donor Program (CMDP). Patients older than 55 years or with a hematopoietic cell transplantation-comorbidity index (HCT-CI) greater than 2 received reduced-intensity conditioning (RIC) regimens, while myeloablative conditioning (MAC) regimens were used for those younger than 55 years. The conditioning regimens are detailed in Fig. 1. Peripheral blood stem cell (PBSC) grafts were mobilized using granulocyte colony-stimulating factor (G-CSF) for 5 days. In the prevention of graft-versus-host disease (GvHD) following haploidentical transplantation, two protocols were utilized: one involving a regimen based on rabbit anti-human thymocyte globulin (ATG) at a dosage of 10 mg/kg (2.5 mg/kg/day on day − 4 to − 1), and the other, a low-dose ATG (2.5 mg/kg/day on day − 2, − 1) plus post-transplant cyclophosphamide (50 mg/kg on day + 3) based regimen, termed the low-dose ATG/PTCy-based regimen [11, 12]. For GvHD prophylaxis after MSD and URD transplantation, a regimen combining cyclosporine (CsA), mycophenolate mofetil (MMF), and short-course methotrexate (MTX) was administered, with an additional 5 mg/kg of ATG when indicated [11]. G-CSF administration commenced on day + 5 and continued until neutrophil engraftment. Prophylactic levofloxacin and acyclovir were initiated at the start of conditioning and continued until hematological reconstitution. Posaconazole prophylaxis was administered from the conditioning until at least three months post-transplant. Cytomegalovirus (CMV) DNA in serum and Epstein-Barr virus (EBV) DNA in whole blood were routinely monitored and preemptive therapy was applied upon reactivation of these viruses [12–14]. Measurable residual disease (MRD) was routinely assessed post-transplantation using two complementary methodologies: multicolor flow cytometry for detecting residual leukemic blasts at a sensitivity threshold of 0.01% (10^−4^), and molecular quantification of disease-specific markers/fusion transcripts via real-time quantitative PCR and/or targeted NGS, with each assay validated to achieve detection limits between 10^−4^ and 10^−5^.

### Prophylactic interventions for relapse

To prevent relapse post-transplant, prophylactic interventions were initiated on 45–60 days post-transplant to patients with poor prognostic characteristics, such as complex cytogenetic abnormalities or TP53 deletion/mutations, as well as those who demonstrated poor disease response prior to transplant and had experienced multiple treatment failures within a short period before undergoing transplantation. The prophylactic intervention regimens included azacitidine alone (32 mg/m^2^/day for 5 days every 28 days for at least 12 cycles), azacitidine combined with venetoclax (400 mg/day for 7 days every 28 days for at least 12 cycles), and azacitidine plus donor-lymphocyte infusion (DLI). Nonetheless, not all eligible patients received these interventions due to factors including clinical judgment, patient compliance, and financial constraints.

### Definition

The engraftment endpoint was based on neutrophil engraftment with absolute neutrophil count (ANC) ≥ 0.5 × 10^9^/L for 3 consecutive days after transplantation without G-CSF. Platelet engraftment was defined as the first of 7 consecutive days with platelet counts of > 20 × 10^9^/L without platelet transfusion. Graft failure was defined as failure of neutrophil engraftment on day 28 following transplantation (primary graft failure, PGF), or loss of donor chimerism after initial engraftment with ≥ 95% recipient cells at any time, not due to relapsed disease (secondary graft failure) [15]. Acute graft-versus-host disease (aGvHD) was diagnosed and graded according to the modified Glucksberg grading of aGvHD [16]and chronic graft-versus-host disease (cGvHD) was diagnosed and graded according to the 2014 National Institutes of Health (NIH) consensus criteria [17].

### Statistical analysis

The Mann–Whitney U test was used for continuous variables and Fisher’s exact test or the χ [2] test was applied for discrete variables. Survival curves were estimated with the Kaplan-Meier method and differences among groups were evaluated by log-rank test. Overall survival (OS) was measured from time of allo-PBSCT until death or censored at time of last patient contact. Recurrence free survival (RFS) was estimated from time of allo-PBSCT to development of AML or death or censored at time of last patient contact, whichever occurred first. GvHD-free, relapse-free survival (GRFS) events were defined according to the original report as the first event among grades III and IV aGvHD, severe cGvHD, relapse, and death. For all patients treated with allo-PBSCT, when estimating non-relapse mortality (NRM), any death in the absence of disease relapse was considered an event. The cumulative incidence of relapse and NRM was estimated by competing risk approach [18]. The Cox proportional hazards model was used for univariable and multivariable analyses. Allo-PBSCT was evaluated as a time-dependent covariate [19]. All P-values reported are two-sided.

##  Results

###  Patient characteristics

**Fig. 2:**
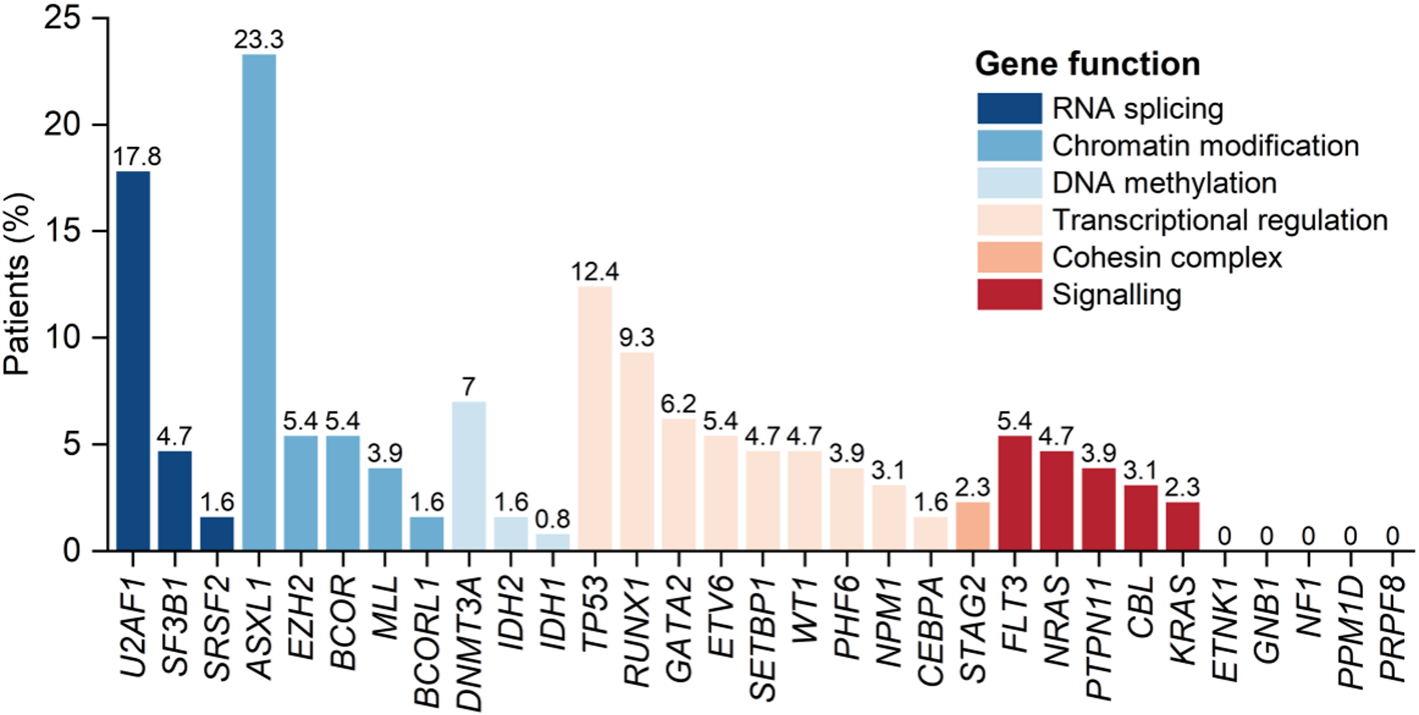
Frequency of each gene mutation. Colors linked to the bars represent the gene function

Five patients were excluded from the total of 134, including 2 with age below 18 years old and 3 with the loss of follow-up. A total of 129 adult patients who underwent transplantation were enrolled into the study. The median age was 45 years (range,18–73 years), and the male was predominant (63.0%). MDS–excess blasts 1/2 (EB1/2) represented 77.5% (100/129) in this cohort. Additionally, 100 patients (77.5%) presented with one or more gene mutations, with 91 patients (70.5%) exhibiting at least one of the 31 IPSS-M related gene mutations, and all the frequency of each gene mutation is shown in Fig. 2. ASXL1 mutation (23.3%) was the most common mutation, followed by U2AF1 (17.8%), TP53 (12.4%), RUNX1 (9.3%) and DNMT3A (7.0%) mutations. The median time from diagnosis to transplantation was 138 days (range, 14-6654 days) for all patients. 69% of patients received haploidentical donors, and 68.2% underwent myeloablative conditioning. The median age of the donors was 32 years (range, 13–62 years), and female donor to male recipient accounted for 20.2%. The median follow-up time was 930 days (range, 23-2712 days). The details of the patient characteristics are presented in Table 1.

**Table 1:** Patient characteristics

Characteristic	Total cohort (*n* = 129)
**Age in years**,** median (IQR)**	45(32–56)
**Sex**,** n (%)**	
Male	72(55.8)
Female	57(44.2)
**WHO category**,** n (%)**	
EB1/EB2	100 (77.5)
MDS-MLD/SLD/RS-MLD	29(22.5)
**IPSS-R**,** n (%)**	
Low	5(3.9)
Intermediate	25(19.4)
High	47(36.4)
Very high	52(40.3)
**IPSS-M categories**,** n (%)**	
Low	2(1.6)
Moderate low	4(3.1)
Moderate high	21(16.3)
High	32(24.8)
Very high **TP53 mutation**,** n (%)**	70(54.3) 16(12.4)
**Treatment before HSCT**,** n (%)**	
HMA	39(30.2)
Chemothraphy	17(13.2)
Others*	73(56.6)
**Conditioning regimens**,** n (%)**	
MAC	88(68.2)
RIC	41(31.8)
**Donor**,** n (%)**	
Haploidentical	89(69.0)
Matched**	40(31.0)
**Time to HSCT in days**,** median (IQR)**	138(74–302)
**Combined umbilical blood**,** n (%)**	
Yes	21(16.3)
No	108(83.7)
Nuclear cells, ×10^7^/kg, median(IQR)	2.8(2.2–3.2)
CD34 + cells, ×10^5^/kg, median(IQR)	1.1(0.6–1.4)
**ABO blood type**,** n (%)**	
**Matched** **Unmatched** Major	64(49.6) 21(16.3)
Minor	37(28.7)
Bidirectional	7(5.4)
**Donor to recipient gender**,** n (%)**	
Female to Male	26(20.2)
Others	103(79.8)
**MNCs**,** ×10**^**8**^**/kg**,** median(IQR)**	15.3 (11.8–18.4)
**CD34**^**+**^ **cells**,** ×10**^**6**^**/kg**,** median(IQR)**	10.7(6.7–14.6)
**CD3**^**+**^ **cells**,** ×10**^**8**^**/kg**,** median(IQR)**	3.5(2.7–4.8)

*Others: include low-dose cytarabine, cyclosporine, danazol, eltrombopag, erythropoietin-stimulating agents, thalidomide, steroid, blood transfusion. **Matched: matched sibling donor (MSD), matched unrelated-donor (URD). WHO, World Health Organization; MDS, myelodysplastic syndromes; MDS-EB1, MDS with excess of blasts type 1; MDS-EB2, MDS with excess of blasts type 2; MDS-MLD, MDS with multilineage dysplasia; MDS-RS-MLD, MDS with ring sideroblasts and multilineage dysplasia; MDS-SLD, MDS with single-lineage dysplasia; IPSS-R, Revised International Prognostic Scoring System; IPSS-M, Molecular International Prognostic Scoring System; MAC, myeloablative conditioning; RIC, reduced-intensity conditioning; HSCT, hematopoietic stem cell transplantation; HMA, hypomethylating agent; MNCs, mononuclear cells

### Transplant outcomes

The engraftment rate was 98.4%, with two patients experiencing PGF. Among the successfully engrafted patients, the median time of neutrophil and platelet engraftment were 12 and 14 days post-transplant, respectively. Within 180 days post-transplant, the cumulative incidences (CIs) of total and grade 2–4 aGvHD were 22.1% and 10.3%, respectively. Within two years after transplantation, the CIs of total and moderate/severe cGvHD were 15.6% and 10.1%, respectively. The details of transplant outcomes are shown in Table 2.

**Table 2:** Transplantation outcomes

Outcomes	Time after HSCT	*N* = 129
Graft failure, n (%)		2(1.6)
Days to ANC engraftment, median (IQR)		12(12–15)
Days to PLT engraftment, median (IQR)		14(12–17)
Acute GvHD, all grades, CI(%)	6 months	22.1
Acute GvHD, grades 2–4, CI(%)	6 months	10.3
Acute GvHD, grades 3–4, CI(%)	6 months	6.4
Chronic GvHD, all grades, CI(%)	2 years	15.6
Chronic GvHD, moderate to severe, CI(%)	2 years	10.1
OS(%)	1 years	81.4
	2 years	76.9
RFS(%)	1 years	79.8
	2 years	75.4
GRFS(%)	1 years	74.4
	2 years	68.1
NRM(%)	1 years	11.6
	2 years	14.7
CIR(%)	1 years	8.6
	2 years	9.9

ANC, absolute neutrophil; PLT, platelet; GvHD, Graft-versus-Host Disease; CI, cumulative incidence; OS, overall survival; RFS, recurrence free survival; GRFS, Graft-versus-Host Disease-Free Recurrence-Free Survival; NRM, non-relapse mortality; CIR, cumulative incidence of relapse

###  IPSS-R and IPSS-M stratification

**Fig. 3:**
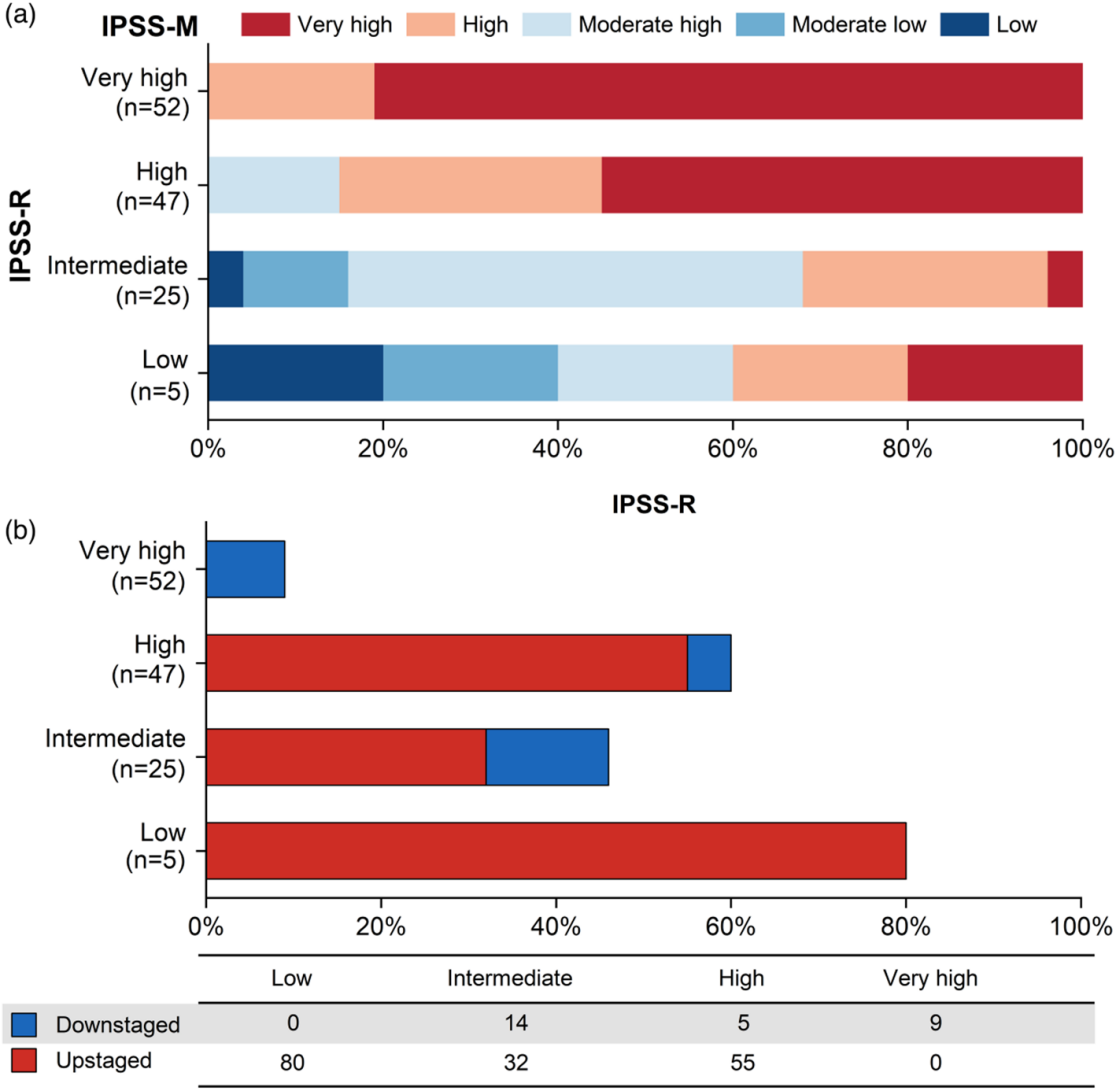
Comparison of the IPSS-M and IPSS-R stratifications.** a** Reclassification of the IPSS-R to IPSS-M. Each row corresponds to one IPSS-R category, and colors represent the IPSS-M categories. **b** Percentage of reclassified patients in each IPSS-R stratum, counting cases with any shift

Patients were stratified into low (3.9%), intermediate (19.4%), high (36.4%), and very high risk (46.3%) categories according to IPSS-R. When the patients were classified based on IPSS-M, 3.1%, 16.3%, 24.8% and 54.2% were defined as moderate low, moderate high, high, and very high risk, respectively, whereas only 2 (1.6%) patients had a low risk (Fig. 3a). To compare the changes of category between IPSS-R and IPSS-M stratifications, moderate low and moderate high risk categories from IPSS-M were combined to an intermediate category. The results showed that about 29.5% of patients in the IPSS-R stratification were upstaged, while 14.0% were downstaged (Fig. 3b).

### **IPSS-R and IPSS-M stratifications for survival**,** CIR and NRM**

**Fig. 4:**
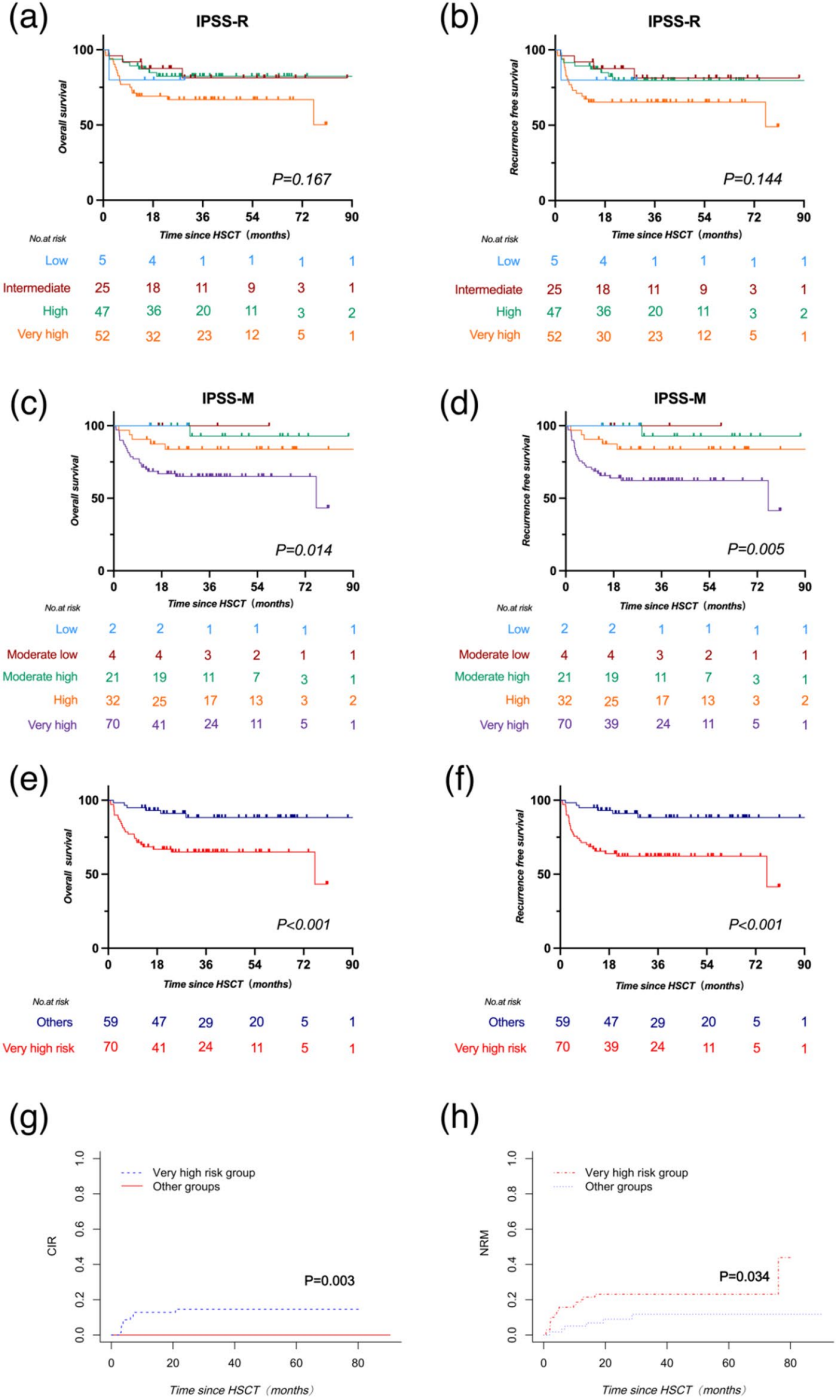
Survival outcomes of Myelodysplastic Syndrome patients based on IPSS-R and IPSS-M Stratifications. **a-b**: overall (OS) and recurrence-free survival (RFS) in IPSS-R. **c-d**: OS and RFS in IPSS-M. **e-f**: OS and RFS for very-high risk versus other categories in IPSS-M. **g-h**: CIR and NRM for very-high risk and other groups in IPSS-M. IPSS-M, Molecular International Prognostic Scoring System; IPSS-R, Revised International Prognostic Scoring System; CIR, Cumulative Incidence of Relapse; NRM, Cumulative Incidence of Non-relapse Mortality, HSCT Hematopoietic stem cell transplantation.

Utilizing the IPSS-R stratification, the OS (*P* = 0.167) and RFS (*P* = 0.144) across risk categories were comparable. Specifically, the 2-year OS rates for the low, intermediate, high, and very-high risk groups were 80.0% (95% CI, 20.4-96.9%), 87.6% (95% CI, 66.3-95.8%), 82.4% (95% CI, 67.8-90.8%), and 66.9% (95% CI, 52.2-78.0%), respectively (Fig. 4a). Corresponding 2-year RFS rates were 80.0% (95% CI, 20.4-96.9%), 87.6% (95% CI, 66.3-95.8%), 79.7% (95% CI, 64.5-89.0%), and 65.3% (95% CI, 50.8-76.5%) (Fig. 4b). Under the IPSS-M framework, 2-year OS and RFS in the very-high risk group were 65.0% (95% CI, 52.4-75.1%) and 62.2% (95% CI, 49.6-72.5%), respectively. Notably, except for the high-risk group with a 2-year OS and RFS of 83.7% (95% CI, 65.2-92.9%) for both, the moderate high, moderate low, and low risk groups showed no relapse or death within two years, culminating in a 2-year OS and RFS of 100%. Two-year OS and RFS in the very-high risk group were significantly lower compared to that in other groups (*P* = 0.014 for OS; *P* = 0.005 for RFS) (Fig. 4c and d).

To analyze the independent factors affecting survival, fourteen variables were sequentially analyzed in a univariate manner. This included gender, age, very high-risk group in IPSS-M, MDS subtype, Karnofsky Performance Status (KPS) score, comorbidity score, intensity of the conditioning regimen, type of transplantation, donor type, and occurrence of aGvHD and cGvHD (Supplementary Table 1). Univariate and multivariate analysis showed that patient age (*P* = 0.009, *P* = 0.017), very-high risk category in IPSS-M (*P* = 0.003, *P* = 0.001), and KPS score (*P* = 0.034, *P* = 0.055) were the independent prognostic factors for OS and RFS (Table 3). When focusing on cumulative incidence of relapse (CIR) post-transplant, only very-high risk category in IPSS-M was an independent risk factor (*P* = 0.003). Consequently, based on the IPSS-M, all patients were stratified into two risk categories: very-high risk and other risk groups. The OS and RFS were significantly lower in the very-high risk group compared to that in the other risk groups (*P* = 0.0006 for OS and *P* = 0.0002 for RFS, respectively) (Fig. 4e, f). Furthermore, considering the competing risks of relapse and mortality, we assessed the CIR and non-relapse mortality (NRM) and found that both CIR (*p* = 0.003) and NRM (*p* = 0.034) were significantly higher in the very-high risk group (Fig. 4g, h). Simultaneously, considering that prophylactic relapse interventions were administered to the subset of patients in the very-high risk group who met the criteria, the overall CIR in this group was not so high. Specifically, within the very-high risk group, patients faced a 2-year CIR of 12.9% (95% CI, 4.95-20.77%), and a 2-year NRM of 21.52% (95% CI, 11.78-31.26%). In stark contrast, the others group exhibited no instances of relapse, yielding a 2-year CIR of 0%, while the 2-year NRM was reported at 9.0% (95% CI, 1.35-16.54%).

**Table 3:** Multivariable analysis for OS and RFS

Factor		95.0% CI			
		Hazard Ratio	Lower	Upper	*P* value
**OS**					
Age (y)	≥ 55	2.577	1.249	5.317	0.010
	< 55				
IPSS-M	Very high risk	3.404	1.382	8.381	0.008
	Others				
KPS (score)	< 90	2.751	1.301	5.815	0.008
	≥ 90				
**RFS**					
Age (y)	≥ 55	2.415	1.187	4.910	0.015
	< 55				
IPSS-M	Very high risk	3.891	1.592	9.507	0.003
	Others				
KPS (score)	< 90	2.387	1.146	4.972	0.020
	≥ 90				

IPSS-M, Molecular International Prognostic Scoring System; KPS, Karnofsky Performance Status; CI, confidence interval; OS, overall survival; RFS, recurrence free survival

### Prophylactic intervention for relapse of patients in very-high risk group with IPSS-M

**Fig. 5:**
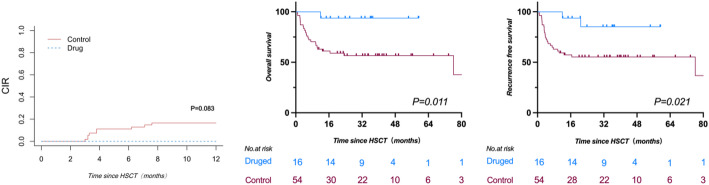
Cumulative incidences of relapse and survival for patients in very-high risk group by IPSS-M with/without the prophylactic interventions. **a** Cumulative incidences of relapse. **b** Overall survival. **c** Recurrence free survival. CIR, Cumulative Incidence of Relapse; HSCT, Hematopoietic stem cell transplantation.

Ten patients (7.8%) experienced hematological relapse post-transplant, all of whom were classified in the very-high risk group according to the IPSS-M stratification system. To elucidate the effect of prophylactic interventions on post-transplant relapse, a separate statistical analysis was conducted on the patients in this subgroup (Supplementary Table 2). Among the 70 patients categorized as very-high risk, 16 underwent prophylactic interventions. These interventions were administered to patients who either displayed poor prognostic features, including complex cytogenetic abnormalities or TP53 deletions/mutations, or who demonstrated inadequate responses to multiple rounds of pre-transplant therapy. Thirteen patients were treated with azacitidine monotherapy ranging for 2 to 14 cycles. Two patients received a combination of azacitidine and venetoclax varying from 1 to 5 cycles. One patient underwent donor lymphocyte infusion (DLI) following azacitidine treatment. Within one year after transplantation, the CIR was 0% in patients with prophylactic interventions, which was lower than the 14.8% (95% CI 5.2%−24.4%) observed in those without (*P* = 0.083, Fig. 5a). The 2-year OS (93.8%, 95% CI 63.2%−99.1%) and RFS (85.2%, 95% CI 51.9%−96.2%) were significantly improved in patients with prophylactic interventions, as opposed to OS (56.6%, 95% CI 42.1%−68.7%, *P* = 0.011) and RFS (55.2%, 95% CI 41.0%−67.4%, *P* = 0.021) in those without interventions (Fig. 5b, c). Meanwhile, we performed a multivariate analysis within the IPSS-M very high-risk cohort for the prognostic factors identified in the entire patient population, including Age, KPS score, and Intervention. The results showed that timely intervention significantly improved patients’ OS (HR = 0.095, *P* = 0.002) and LFS (HR = 0.177, *P* = 0.002).(Supplementary Table 3).

## Discussion

To improve the survival outcomes post-transplant for MDS patients, the implementation of a more accurate risk scoring system and timely clinical interventions is imperative. Our research suggested that, compared to IPSS-R, the IPSS-M system significantly improved the prognostic stratification for MDS patients undergoing allo-PBSCT. Furthermore, early prophylactic interventions for relapse, based on the IPSS-M system, could reduce the risk of relapse and improve survival for patients in the very-high risk category.

Mounting evidences suggest that the inclusion of mutational data optimizes prognostic classification for MDS patients [20, 21]. The newly developed IPSS-M by the IWGPM retains the cytogenetic classification of IPSS-R [3, 22]while incorporates binary features of 16 main effect genes and a number of mutations from a residual group of 15 genes at the molecular level. Key molecular mutations, including multi-hit TP53 and FLT3-ITD/TKD, play a pivotal role in predicting adverse outcomes. This personalized prognostic model is more precise, resulting in nearly half (46%) of patients being reclassified from their original IPSS-R categories. Similarly, in our study cohort, 43.5% of patients were reclassified, predominantly with an upward adjustment in risk rating (29.5%). Among these reclassified patients, 62% had detectable mutations in one or more IPSS-M-related genes. However, our study’s distribution of risk categories within the IPSS-M diverged from previously reported actual MDS cohorts without HSCT [6]showing a skew towards higher risk and very-high risk categories, which may be associated with the eligibility criteria for HSCT.

In the study by Sandra Novoa Jáuregui et al. [23]99 patients (15.2%) could potentially become new candidates for altered therapeutic approaches following reclassification, with advanced age (> 70 years) and/or multiple comorbidities precluding 36 patients from receiving allogeneic HSCT. The remaining 63 patients (9.7%) may derive clinical benefits from the reclassification according to IPSS-M. Similarly, the research conducted by Elisabetta Sauta et al. [7] found that, among MDS patients treated with HSCT, the IPSS-M significantly enhanced the predictive ability for OS probabilities, particularly effective in capturing the likelihood of relapse, aligning with our study findings. In our cohort, post-transplant survival and relapse were substantially correlated with the IPSS-M risk categories. Patients classified within the very-high risk group exhibited markedly reduced OS and RFS, with concurrent increases in CIR and NRM. Conversely, among the low, moderate low, and moderate high-risk categories, there were no instances of death or relapse within two years. These findings also show a degree of consistency with another study from China involving MDS patients undergoing HSCT [24].

Allo-HSCT remains the only curative option for patients with high-risk MDS. Advances in preparative regimens and supportive care have reduced treatment-related mortality and expanded the role of transplantation, enabling a greater number of patients to undergo HSCT [25]. Moreover, transplantation performed early after the diagnosis is associated with the most favorable transplant outcomes [26]suggesting that patients identified as higher risk by the IPSS-M should be considered for transplantation earlier than those stratified by conventional scoring systems (IPSS-R) [27, 28]. However, post-transplant relapse continues to be a predominant cause of morbidity and mortality in MDS, presenting a significant clinical challenge in identifying such patients and implementing timely interventions to prevent relapse. While the overall incidence of post-transplant relapse in our cohort was relatively low at 7.8%, all relapse cases were concentrated within the very-high risk group as defined by the IPSS-M model. This finding underscores the model’s strong predictive ability for identifying patients most susceptible to relapse. Our study revealed that patients in the very-high risk group had a poorer survival prognosis. Nevertheless, the data also demonstrated the effectiveness of proactive post-transplant interventions in reducing the likelihood of relapse for these high-risk individuals. By implementing tailored pharmacotherapy protocols and donor lymphocyte infusion, we have markedly reduced the likelihood of relapse and mortality. In our intervention cohort, the absence of relapse among patients during the initial year post-transplant is a testament to the potential for significantly improved survival outcomes. Despite its potential utility as a supplementary tool for the clinical management of MDS patients, it must be acknowledged that the IPSS-M currently has several limitations, given that it was developed within a cohort largely naive to disease-modifying treatments. To better guide timely interventions post-transplant for MDS patients, there is a pressing need for prospective clinical trials focused on post-transplant relapse, in conjunction with the validation of measurable residual disease (MRD) methodologies.

There are limitations in our study. Firstly, our cohort primarily consisted of patients who underwent HSCT with a MAC conditioning regimen, warranting caution when generalizing our findings to other cohorts, such as chemotherapy-only populations. Additionally, due to the cost and limited application of NGS, molecular testing is not routine for all MDS patients, and we noted that genes such as ETNK1, GNB1, NF1, PPM1D, and PRPF8 were not expressed in our patients, which cannot exclude the possibility of data omission due to the nature of a single-center retrospective study. However, studies have shown that even with a small amount of missing molecular feature data, the IPSS-M model retains strong predictive power [7]. We also focused on molecular mutations at the initial diagnosis, and due to cost constraints, many patients could not monitor mutation information as treatment progressed, including at initial remission, pre-transplant, post-engraftment, etc. Whether the dynamic changes in gene mutation frequency can provide guidance for prognostic prediction and treatment choice remains an area for further research. Furthermore, the intrinsic heterogeneity of MDS, the spectrum of post-transplant complications, and the diverse drug responses among patients complicate the standardization of prophylactic intervention strategies, potentially influencing the interpretation of prognostic outcomes.

In summary, our study suggests that compared to IPSS-R, the IPSS-M model has better prognostic discrimination ability in MDS patients receiving PBSCT. Furthermore, we should pay more attention to patients in the very-high risk category of this model to lay the groundwork for clinical decisions on whether to intervene aggressively after transplantation.

## Electronic supplementary material

Supplementary Material 1

## Data Availability

The data that support the findings of this study are available from the corresponding author upon reasonable request.

## Declarations

### Guidelines

The study protocol adhered to the Declaration of Helsinki principles.
