# Supplementary material for: IPSS-M outperforms IPSS-R in prognostic stratification and guides effective interventions for very High-Risk myelodysplastic syndrome patients undergoing allogeneic hematopoietic stem cell transplantation
Source: Discov Oncol. 2025 Jul 11;16:1315. doi: 10.1007/s12672-025-03155-1 (PMC12254102; doi:10.1007/s12672-025-03155-1)
Supplement: Supplementary file 1 — Supplementary Material 1 [file 12672_2025_3155_MOESM1_ESM.docx]

**Supplementary Table 1.Univariate analysis for OS and RFS**

| Factor | | 95.0% CI | | | |
| --- | --- | --- | --- | --- | --- |
|  |  | Hazard Ratio | Lower | Upper | P value |
| **OS** | |  | | | |
| Age (y) | ≥55 | 2.815 | 1.388 | 5.709 | 0.004 |
|  | <55 |  |  |  |  |
| IPSS-M | Very high risk | 4.173 | 1.711 | 10.181 | 0.002 |
|  | Others |  |  |  |  |
| KPS (score) | <90 | 3.405 | 1.622 | 7.155 | 0.001 |
|  | ≥90 |  |  |  |  |
| Acute GvHD (grades 3-4)  Sexual  Type（MDS）  Combined umbilical blood  HCT-CI（score）  Conditioning regimen intensity  Haploidentical  Age（Donor）（y）  Sexual（Donor）  ABO incompatible  Chronic GVHD | Yes | 3.474 | 1.321 | 9.133 | 0.012 |
|  | No  Male  Female  EB1/EB2  Others  Yes  No  ≥1  0  RIC  MAC  Yes  No  ≥50  <50  F to M  Others  Yes  No  <55  ≥55 | 1.083  1.246  0.635  2.943  2.457  1.665  1.100  1.943  1.043  0.599 | 0.526  0.511  0.215  0.401  1.211  0.717  0.384  0.903  0.516  0.243 | 2.230  3.041  1.871  21.608  4.981  3.869  3.153  4.183  2.110  1.476 | 0.828  0.629  0.410  0.289  0.013  0.236  0.859  0.089  0.906  0.266 |
| **RFS** | |  |  |  |  |
| Age (y) | ≥55 | 2.544 | 1.279 | 5.062 | 0.008 |
|  | <55 |  |  |  |  |
| IPSS-M | Very high risk | 4.610 | 1.902 | 11.171 | 0.002 |
|  | Others |  |  |  |  |
| KPS (score) | <90 | 3.051 | 1.472 | 6.326 | 0.003 |
|  | ≥90 |  |  |  |  |
| Acute GvHD (grades 3-4) | Yes | 3.319 | 1.269 | 8.680 | 0.014 |
|  | No |  |  |  |  |
| Sexual  Type（MDS）  Combined  umbilical blood  HCT-CI（score）  Conditioning regimen intensity  Haploidentical  Age（Donor）（y） | Male  Female  EB1/EB2  Others  Yes  No  ≥1  0  RIC  MAC  Yes  No  ≥50  <50 | 0.942  1.227  0.591  3.207  2.210  1.249  1.330 | 0.470  0.552  0.202  0.413  1.111  0.601  0.512 | 1.887  3.242  1.732  22.181  4.935  2.788  3.456 | 0.866  0.520  0.338  0.276  0.020  0.510  0.558 |
| Sexual（Donor） | F to M | 1.800 | 0.846 | 3.834 | 0.127 |
|  | Others |  |  |  |  |
| ABO incompatible  Chronic GVHD | Yes  No  <55  ≥55 | 1.197  0.599 | 0.603  0.243 | 2.347  1.476 | 0.608  0.266 |

IPSS-M, Molecular International Prognostic Scoring System; KPS, Karnofsky Performance Status; GvHD, Graft-versus-Host Disease; CI, confidence interval; OS, overall survival; RFS, recurrence free survival; HCT-CI, Hematopoietic Cell Transplantation-Comorbidity Index.

**Supplementary Table 2. Patient characteristics between intervention and non-intervention groups**

| **Characteristic** | **Intervention group(n=16)** | **Non-intervention group(n=54)** | **P value** |
| --- | --- | --- | --- |
| **Age in years, median (range)** | 48(21-64) | 48(18-71) | 0.716 |
| **Sex, n (%)** |  |  |  |
| Male | 11(68.8) | 27(50.0) | 0.494 |
| Female | 5(31.2) | 27(50.0) | 0.598 |
| **WHO category, n (%)** |  |  |  |
| EB1/EB2 | 15(93.8) | 50(92.6) | 1.000 |
| MDS-MLD/SLD/RS-MLD | 1(6.3) | 4(7.4) | 1.000 |
| **Treatment before HSCT, n (%)** |  |  |  |
| HMA | 5(31.2) | 21(38.9) | 0.788 |
| Chemothraphy | 2(12.5) | 7(13.0) | 1.000 |
| Others* | 9(56.3) | 26(48.1) | 0.810 |
| **Conditioning regimens, n (%)** |  |  |  |
| MAC | 11(68.8) | 34(63.0) | 1.000 |
| RIC | 5(31.2) | 20(37.0) | 1.000 |
| **Donor, n (%)** |  |  |  |
| Haploidentical | 9(56.3) | 35(54.8) | 0.820 |
| Matched** | 7(43.8) | 19(35.2) | 0.789 |
| **Time to HSCT in days, median (IQR)** | 45(90-391) | 111(65-184) | 0.634 |
| **Combined umbilical blood, n (%)** |  |  |  |
| Yes | 1(6.3) | 7(13.0) | 0.678 |
| No | 15(93.8) | 47(87.0) | 1.000 |
| Nuclear cells，×10^7^/kg，median（range） | 2.6(2.6-2.6) | 1.5(0.9-1.8) | 0.180 |
| CD34^+^ cells，×10^5^/kg，median（range) | 1.1(1.1-1.1) | 0.8(0.7-1.2) | 1.000 |
| **ABO blood type，n (%)** |  |  |  |
| **Matched**  **Unmatched**  Major | 6(37.5)  1(6.3) | 27(50.0)  9(16.7) | 0.406  0.406 |
| Minor | 7(43.8) | 15(27.8) | 0.238 |
| Bidirectional | 2(12.5) | 3(5.6) | 0.321 |
| **Donor to recipient gender，n (%)** |  |  |  |
| Female to Male | 4(25.0) | 13(24.1) | 1.000 |
| Others | 12(75.0) | 41(75.9) | 1.000 |
| **MNCs, ×10^8^/kg, median（IQR）** | 15.0(12.8-16.4) | 16.3(13.2-21.3) | 0.275 |
| **CD34^+^ cells, ×10^6^/kg, median（IQR）** | 11.2(8.4-16.1) | 10.3(6.7-14.4) | 0.397 |
| **CD3^+^ cells , ×10^8^/kg, median（IQR）** | 3.0(2.7-3.9) | 3.4(2.6-4.5) | 0.612 |

*Others: include low-dose cytarabine, cyclosporine, danazol, eltrombopag, erythropoietin-stimulating agents, thalidomide, steroid, blood transfusion.

**Matched: matched sibling donor (MSD), matched unrelated-donor (URD)

WHO, World Health Organization; MSD, myelodysplastic syndromes; MDS-EB1, MDS with excess of blasts type 1; MDS-EB, MDS with excess of blasts type 2; MDS-MLD, MDS with multilineage dysplasia; MDS-RS-MLD, MDS with ring sideroblasts and multilineage dysplasia; MDS-SLD, MDS with single-lineage dysplasia; MAC, myeloablative conditioning; RIC, reduced-intensity conditioning; HSCT, hematopoietic stem cell transplantation; HMA, hypomethylating agent; MNCs, mononuclear cells.

**Table S3. Multivariable analysis for OS and RFS**

| Factor | | 95.0% CI | | | |
| --- | --- | --- | --- | --- | --- |
|  |  | Hazard Ratio | Lower | Upper | P value |
| **OS** | |  | | | |
| Age (y) | ≥55 | 3.870 | 1.679 | 8.919 | 0.001 |
|  | <55 |  |  |  |  |
| KPS (score) | <90 | 2.323 | 1.121 | 4.753 | 0.024 |
|  | ≥90 |  |  |  |  |
| Intervention | yes | 0.095 | 0.013 | 0.710 | 0.002 |
|  | no |  |  |  |  |
| **RFS** | |  |  |  |  |
| Age (y) | ≥55 | 3.318 | 1.498 | 7.349 | 0.003 |
|  | <55 |  |  |  |  |
| KPS (score) | <90 | 2.651 | 1.325 | 5.227 | 0.013 |
|  | ≥90 |  |  |  |  |
| Intervention | yes | 0.177 | 0.041 | 0.76 | 0.002 |
|  | no |  |  |  |  |
|  |  |  |  |  |  |

IPSS-M, Molecular International Prognostic Scoring System; KPS, Karnofsky Performance Status; CI, confidence interval; OS, overall survival; RFS, recurrence free survival.
